# Interaction of TX-100 and Antidepressant Imipramine Hydrochloride Drug Mixture: Surface Tension, ^1^H NMR, and FT-IR Investigation

**DOI:** 10.3390/gels8030159

**Published:** 2022-03-04

**Authors:** Malik Abdul Rub, Naved Azum, Dileep Kumar, Abdullah M. Asiri

**Affiliations:** 1 Center of Excellence for Advanced Materials Research, King Abdulaziz University, Jeddah 21589, Saudi Arabia; nhassan2@kau.edu.sa (N.A.); aasiri2@kau.edu.sa (A.M.A.); 2 Chemistry Department, Faculty of Science, King Abdulaziz University, Jeddah 21589, Saudi Arabia; 3 Division of Computational Physics, Institute for Computational Science, Ton Duc Thang University, Ho Chi Minh City 700000, Vietnam; 4 Faculty of Applied Sciences, Ton Duc Thang University, Ho Chi Minh City 700000, Vietnam

**Keywords:** amphiphilic drug, nonionic surfactant, surface property, thermodynamic, chemical shift, FT-IR

## Abstract

Interfacial interaction amongst the antidepressant drug-imipramine hydrochloride (IMP) and pharmaceutical excipient (triton X-100 (TX-100-nonionic surfactant)) mixed system of five various ratios in dissimilar media (H_2_O/50 mmol·kg^−1^ NaCl/250 mmol·kg^−1^ urea) was investigated through the surface tension method. In addition, in the aqueous solution, the ^1^H-NMR, as well as FT-IR studies of the studied pure and mixed system were also explored and deliberated thoroughly. In NaCl media, properties of pure/mixed interfacial surfaces enhanced as compared with the aqueous system, and consequently the synergism/attractive interaction among constituents (IMP and TX-100) grew, whereas in urea (U) media a reverse effect was detected. Surface excess concentration (*Γ_max_*), composition of surfactant at mixed monolayer ($X_{1}^{\sigma }$), activity coefficient (*f*_1_^σ^ (TX-100) and *f*_2_^σ^ (IMP)), etc. were determined and discussed thoroughly. At mixed interfacial surfaces interaction, parameter (*β*^σ^) reveals the attractive/synergism among the components. The Gibbs energy of adsorption ($\Delta G_{\mathrm{ads}}^{o})$ value attained was negative throughout all employed media viewing the spontaneity of the adsorption process. The ^1^H NMR spectroscopy was also employed to examine the molecular interaction of IMP and TX-100 in an aqueous system. FT-IR method as well illustrated the interaction amongst the component. The findings of the current study proposed that TX-100 surfactant could act as an efficient drug delivery vehicle for an antidepressant drug. Gels can be used as drug dosage forms due to recent improvements in the design of surfactant systems. Release mechanism of drugs from surfactant/polymer gels is dependent upon the microstructures of the gels and the state of the drugs within the system.

## 1. Introduction

Gels are used for various applications based on their drug-loading properties, rheological properties, and release mechanisms. Drugs can either be soluble in water with no interaction through any of the constituents, electrostatically/hydrophobically tied with polymer, or soluble within micelles and polymer/surfactant associates. The use of surfactant/polymer systems for gene therapy has a great deal of promise, and certain polymers can interact with the natural (nonionic) surfactant, which can be utilized to lock in bile salts for controlling cholesterol levels in the body. The interfacial/micellar characteristics of amphiphiles mixtures have been broadly studied due to their extensive applications, for instance, hydrate inhibitors, biologicals, foaming, in fabric moderating, pharmaceutics, improved oil recovery procedure, and so forth [1,2,3]. In aqueous/non-aqueous solvent, the surfactant monomers (comprising hydrophobic and hydrophilic parts into single molecules) were orientated into an associated form after surpassing a certain concentration into the solution (solvent) and formed the associate structure, called the micelle. The corresponding concentration is symbolized as the critical micelle concentration (*cmc*) [3,4,5,6]. Surfactant micelles revealed a considerable role in the solubilization of several hydrophobic materials including drugs [3,7]. Surfactant also acts as a drug carrier in combination with a specific additive, and therefore, extensive inspections of the influences of several additives (organic and inorganic) on the association performance of the drug are needed [3,7]. As compared with singular surfactant micelle formation, the mixed surfactants have substantial considerable properties in a variety of features [3]. Usually, a mixed surfactants system (ionic amphiphile with other ionic or nonionic amphiphiles) has smaller surface energy, higher solubilization capability, and smaller *cmc* along with higher surface activities as compared to the singular surfactants because of the attractive interaction/synergetic influence [3,8]. To diagnose osteoarthritis, Yin et al. [9] have made significant progress in eliminating major hurdles to using extracellular vesicles for delivery and as markers. Osteoarthritis therapeutics can be delivered effectively via extracellular vesicles because of their size, surface expression patterns, low immunogenicity, and low cytotoxicity.

Within various kinds of surfactants (cationic/anionic/nonionic), the non-ionic surfactant is valued as the best one for safe drug delivery, as they are physiologically more supportable than ionic surfactant [7]. TX-100 is one of the most applied surfactants in bio-chemical and chemical practices. The head groups of non-ionic surfactants consist of no electrical charge; therefore, they are generally soluble in water through H-bonding formation between the hydrophilic parts of the surfactant with water. Triton X-100 (TX-100) non-ionic surfactant has a huge industrial significance applied in the formulation of foams and found several applications in the pharmaceutical sciences for purpose of cleaning and as an ingredient in a few curative products [10,11]. TX-100 comprises a hydrophilic chain of 9 to 10 ethylene oxide units coupled with an aromatic ring, having a branched hydrocarbon chain. Different properties (interfacial, micellization, drugs solubilization ability, clouding property, etc.) of TX-100 in the occurrence of charge amphiphiles have been analyzed by means of experimental methods [3,12,13]. TX-100 varies from other conventional nonionic surfactants because their hydrophilic portion was found to be longer compared with the hydrophobic section of the monomer [14]. Herein, the interaction of TX-100 with antidepressant IMP was evaluated by means of different techniques. The mixed system of IMP+TX-100 reveals a compact packing at the surface as well as higher interfacial activity.

At a higher concentration, numerous amphiphilic drugs also formed a micellar structure in a similar manner to a conventional surfactant [15,16]. Pure amphiphilic drugs self-association studies, for any particular purpose are usually out-of-focus due to their high *cmc*, because of the use of a high amount of a drug, which might create numerous side effects [17]. Therefore, amphiphilic drugs are generally used in combination with additives such as surfactant, hydrotropes, bile salts, etc., as a drug carrier that generally forms mixed micelles [8,15]. As a mixture, the *cmc* value reduced more than 10 times. Hence, a very low quantity of drug is used along with a mixed micellar system to raise the absorption of numerous drugs [8].

**Scheme 1:**
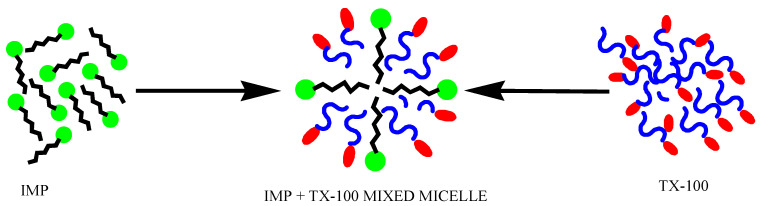
Mixed micelle formation of the IMP+TX-100 mixed system.

Imipramine hydrochloride (IMP) is an amphiphilic tricyclic antidepressant drug that has two main parts, one is a large rigid tricyclic hydrophobic ring (tail) and the other one is a small alkyl amine part (head) and endures aggregation but higher concentration [15]. This drug color is white to off-white, odorless compound, and is employed to treat depression. The nature of IMP drug is protonated (cationic) at a lower pH range (below 7) and deprotonated at a high range (above 7) of pH (p*K*_a_ = 9.5) [15]. Apart from their uses to heal depression, this drug also indicated some unwanted impact. Consequently, to lessen the unwanted impact of IMP, mixed micellization investigation of IMP with TX-100 (as a drug carrier) (Scheme 1) was conducted in different media by means of the several methods.

Previously, our group have examined solution (bulk) properties (mixed micellization behavior) of pure and mixed system of IMP and TX-100 in water, NaCl, and urea media [18] and the current study is an extension of our previous work [18]. Herein, the interfacial properties of IMP and TX-100 mixture were evaluated by tenstiometic method in different media, along with ^1^H NMR and FT-IR spectroscopy, which were also employed to evaluate the interaction amongst IMP and TX-100 in an aqueous system. Combining IMP with TX-100, might enhance drug characteristics, such as their solubility along with stability in living atmospheres [8,19]. Previously, in an aqueous solution, Alam and Siddiq [20] examined the association and surface behavior of an IMP drug and TX-100 mixed system by differing the mole fraction of a drug by tensiometric method. Irrespective of surface tension and ^1^H NMR methods, the FTIR study of the akin system in aqueous media was also investigated to crisscross the reliability of the interaction between IMP drug and TX-100. ^1^H NMR of IMP+TX-100 mixture in five different ratios has been investigated to explain the mechanism of IMP and TX-100 interactions. Several theoretical models regarding the interfacial behavior are employed to illustrate the mixed monolayer formation of the drug-surfactant mixed system in three different media. Various parameters, such as surface excess concentration (*Γ_max_*), composition of constituent at mixed monolayer and the interaction parameter (*β*^σ^) at interface, activity coefficient of employed ingredients (*f*_1_^σ^ (TX-100) and *f*_2_^σ^ (IMP)) at the boundary, packing parameter, etc., at the mixed monolayer, have been assessed and discussed [3,21]. Different thermodynamic functions (Gibbs’s energy of adsorption ($\Delta G_{\mathrm{ad}}^{o}$), minimum free energy (*G_min_*), excess free energy at mixed monolayer ($\Delta G_{\mathrm{ex}}^{\sigma }$)), and chemical shifts by ^1^H NMR study have also been thoroughly evaluated and debated. According to the current study, the results have relevance to model drug delivery, but no direct evidence can be drawn for drug delivery. As a result of this study, drugs and their possible carriers are examined physiochemically using various theoretical models, which is vital since the surfactant may also be utilized as a drug carrier. In addition, the choice of 50 mmol·kg^−1^ NaCl and 250 mmol·kg^−1^ urea concentration was not based on any specific reason other than to examine the effects of salt and urea that are normally found in human being. To provide knowledge (thermodynamic and additional) for the widely used drug-surfactant combinations in the absence and presence of NaCl and urea in drug delivery, our primary goal had been to exhibit how the two ingredients interacted in the aqueous system as well as in salt and urea media. Further enhancement of drug-surfactant conjugate delivery systems is possible if salt/urea are present as their presence increases/decreases the spontaneity of the mixture.

## 2. Results and Discussion

### 2.1. Characteristics at the Air-Interfacial Surfaces of Pure and Mixed System

Amphiphiles are likely to settle at the air-interfacial surface as compared with the bulk solution. Gibbs’s adsorption equation [22] is employed to assess a variety of surface parameters of drug–surfactant mixed system. All interfacial parameters were evaluated by using the surface tension plot given in our previous work [18]. The adsorbed quantity of molecules in each unit area of the surface is computed through the assistance of Gibbs adsorption equation [22]. The surface excess concentration (*Γ_max_*) along with minimum area per monomer (*A_min_*) values in aqueous/non-aqueous media were determined utilizing the subsequent equations [3,22]:

(1) $$\Gamma_{max}=-\frac{1}{2.303nRT}{\left(\frac{\partial \gamma }{\partial log\left(C\right)}\right)}_{}\;(\mathrm{mol}\cdot m^{-2}),$$

(2) $$A_{min}=\frac{10^{20}}{N_{A}\;\Gamma_{max}}\;({Å}^{2}).$$

Here, the *γ*, *C*, *T*, *n*, *R*, and *N_A_* is the surface tension (mN·m^−1^), employed concentration of IMP, TX-100, or IMP+TX-100 mixtures, temperature, whole number of solute species obtained during adsorption, gas constant, and Avogadro number, respectively [3]. The *n* is considered 2 and 1 in the case of individual IMP and TX-100, respectively. However, in mixtures, *n* values were assessed using term: $n=n_{1}X_{1}^{\sigma }+n_{2}\left(1-X_{1}^{\sigma }\right)$ [3], where *n_1_* = number of species in component 1 and *n_2_* = number of species in component 2 after ionization. $X_{1}^{\sigma }$ = interfacial composition of component 1 at the mixed surface (Table 1). Throughout the study, the first component, or component 1, is used for TX-100 and the second component, or component 2, is used for IMP. The slope = *∂γ*/∂*log*(*C*) value is attained from the *γ* vs. *log*(*C*) plot of any fixed concertation in all cases.

In the ideal state, the minimum surface area per molecule ($A^{id}$) was evaluated by means of Equation (3):

(3) $$A^{id}=X_{1}^{\sigma }A_{1}+(1-X_{1}^{\sigma })A_{2}.$$

Here, *A*_1_ and *A*_2_ = per monomer minimum head group area of surfactant and IMP correspondingly. The assessed *Γ_max_*, $A_{min}$ and $A^{id}$ value of individual and mixed components (IMP, TX-100, and IMP+TX-100) in the existence of different media were revealed in Table 1.

Table 1 showed the value of *Γ_max_* and $A_{min}$ of individual TX-100 in the aqueous system, which was found to be 36.02 mol m^−2^ and 46.10 Ǻ^2^ respectively, revealing that their value is in the same range with the previously reported value [23]. The parameter *A*_min_ value showed the opposite trend with the *Γ_max_* value means, as each parameter was in reverse with each other. The *Γ_max_* value of singular IMP obtained lesser than the *Γ_max_* value of pure TX-100 means, and the *A_min_* value showed the opposite behavior. This obtained behavior viewed that TX-100 molecules favored a compacted or strongly packed arrangement at the air-solvent interface as compared with IMP regardless of the media used, and therefore TX-100 showed more surface activity. The value of *Γ_max_* of the IMP+TX-100 mixed system was found above the *Γ_max_* value of singular IMP but was obtained below the *Γ_max_* value of TX-100, so we can observe that mixed system surface activity was found higher than pure IMP but less than pure TX-100. In an aqueous system, the *Γ_max_* value of the IMP+TX-100 mixed system was found to increase with an increase in *α*_1_ of TX-100, observing that the mixed system surface activity increases with the increase of the composition of TX-100 in the solution mixture. However, in the presence of NaCl or U, the *Γ_max_* value of the IMP+TX-100 mixture has not viewed a specific trend, nor did *A_min_*, since *A_min_* is inversely proportional to *Γ_max_*.

The *Γ_max_* value in NaCl media of IMP+TX-100 mixtures was achieved higher than other employed media (H_2_O or U). The electrostatic repulsions between the ingredient’s monomers decreased in NaCl media, observing that the efficiency of the molecules′ existence at the interfacial surface increased and high compactness of IMP+TX-100 mixtures existed. However, in U solvent, pure IMP, and TX-100, *Γ_max_* value found less but does not show any proper trend for mixed system.

**Figure 1:**
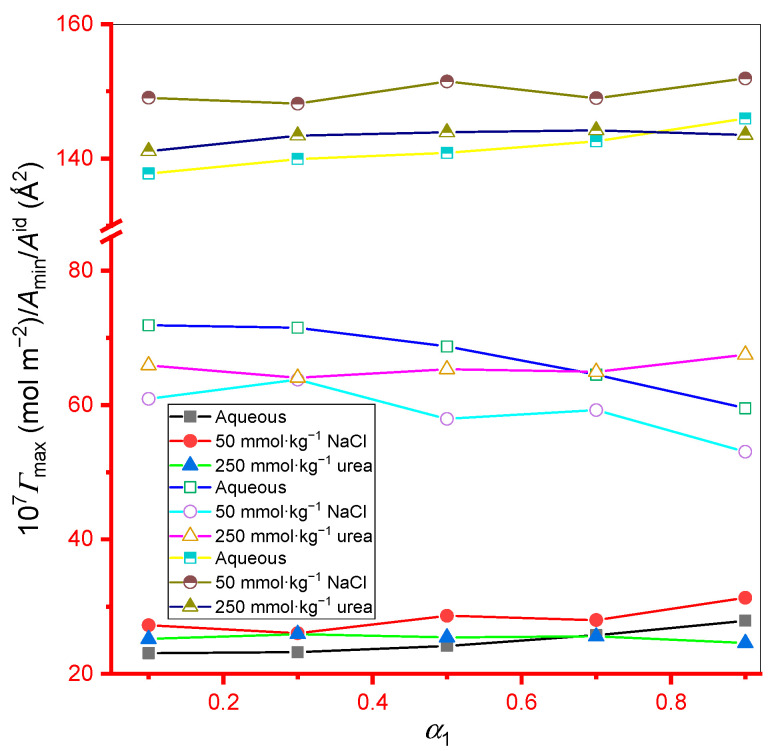
Plot of *Γ_max_*/*A_min_*/*A^id^* against α_1_ plot for IMP+TX-100 mixture in different media (filled, open, and half-filled symbols represent *Γ_max_*, *A_min_*, and *A^id^*, respectively).

The $A_{}^{id}$ value of IMP+TX-100 mixtures were observed to be higher than experimental $A_{min}$, implying that the space taken by apiece monomers was found below as expected for their ideal behavior. For mixtures (IMP+TX-100), the $A_{min}$ value was obtained below the value of $A_{min}$ of pure IMP. This result indicates that the introduction of TX-100 in the solution of IMP causes decreases in the repulsive force between IMP monomer molecules, and hence the value of mixture $A_{min}$ decreased. Figure 1 showed the *Γ_max_*/*A_min_*/*A^id^* vs. α_1_ plot for IMP+TX-100 mixture in diverse media (filled, open, and half-filled symbols represent *Γ_max_*, *A_min_*, and *A^id^*, respectively), which shows the comparison of different surface parameters graphically.

The parameter surface tension value at the *cmc*, is symbolized via *γ_cmc_* and the obtained value is depicted in Table 1. The *γ_cmc_* value of singular and mixed system (IMP+TX-100) in aqueous and NaCl media were taken from the graph of our group’s earlier published work [18]. For individual TX-100 and IMP+TX-100 mixtures, the value of *γ_cmc_* was found close to each other’s means in the same range, irrespective of the solvent employed. However, their value for individual IMP was found quite higher. The surface parameters-surface pressure at the *cmc* (*π_cmc_*) and the adsorption efficiency, i.e., *pC*_20_, was also exploited for individual ingredients and the IMP+TX-100 mixtures in all media. At the *cmc,* the surface pressure (*π_cmc_*) parameter was explored by means of Equation (4) [3].

*π_cmc_* = (*γ*_0_ − *γ_cmc_*). (4)

In Equation (4), *γ*_0_ signified the pure solvent surface tension, and *γ_cmc_* indicated the *γ* at the *cmc* of the single and mixed components. All assessed values of *γ_cmc_* and *π_cmc_* are presented in Table 1. The obtained *π_cmc_* is lowest for IMP irrespective of the media utilized but was found to be close to each other for individual TX-100 and the IMP+TX-100 mixture [24].

Another parameter, called *pC*_20_, allowed the adsorption efficiency of the constituents at the interfacial surface. This parameter is demarcated as the negative logarithm of the concentration of monomer(s), as the individual solvent surface tension is lessened by 20 mN m^−1^ (*C*_20_) [3]:

*pC*_20_ = −*logC*_20_. (5)

The higher the *pC*_20_ value, the larger the amphiphile efficiency for adsorption (higher surface activities) because a smaller amount (volume) of prepared solutions is needed to condense the solvent surface tension by 20 mN·m^–1^. The obtained value of *pC*_20_ of IMP was considerably lower to a large extent, as compared to *pC*_20_ achieved for individual TX-100 regardless of the solvent used, which again confirmed that the IMP drug was less surface-active as compared with TX-100 (Table 1). This obtained phenomena showed that TX-100 has better adsorption ability along with being more effective in surface tension reduction of the solvent [24]. The *pC*_20_ value for IMP+TX-100 mixed systems was higher than individual IMP, observing that the mixed systems were more surface-active as compared with IMP, and their value increased with an enhancement in *α*_1_ of TX-100, but blended systems *pC*_20_ value was found near the *pC*_20_ value TX-100 (Table 1). Pure species, as well as IMP+TX-100 mixtures *pC*_20_ value enhanced in the existence of NaCl because of the better surface activity in NaCl media as compared with aqueous system and the reverse trend, which was detected in the existence of U (Table 1).

### 2.2. Composition of Component and Interaction Parameters at the Air-Interfacial Surfaces

Before the start of micellization, at the interfacial surface, a mixed monolayer formation took place through adsorption phenomena. Rosen′s theory [25] was applied to assess the composition of the constituent at mixed monolayer as well as the interaction parameter (*β*^σ^) at the interface through subsequent equations.

(6) $$\frac{{\left(X_{1}^{\sigma }\right)}^{2}\ln\left(\alpha_{1}C/X_{1}^{\sigma }C_{1}\right)}{{\left(1-X_{1}^{\sigma }\right)}^{2}\ln\left[\left(1-\alpha_{1}\right)C/\left(1-X_{1}^{\sigma }\right)C_{2}\right]}=1,$$

(7) $$\beta^{\sigma }=\frac{\ln\left(\alpha_{1}C/X_{1}^{\sigma }C_{1}\right)}{{\left(1-X_{1}^{\sigma }\right)}^{2}}.$$

In Equation (6), the $X_{1}^{\sigma }$ = composition of the surfactant in the mixed monolayer (IMP+TX-100), and in Equations (6) and (7) *C*_1_ = TX-100 concentration (first component), *C*_2_ = IMP concentration (second component), and *C* = mixed monolayer concentration (IMP+TX-100) at different *α*_1_, which is used for lessening the surface tension of a solvent of any selected value for all cases (pure and mixture).

The assessed values of $X_{1}^{\sigma }$ and *β^σ^* of all systems are itemized in Table 1. Herein, the $X_{1}^{\sigma }$ (TX-100 composition at the mixed component surface) values were obtained amid 73% to 92% in all studied media, displaying that mainly TX-100 comprises the mixed monolayer. By increasing the *α*_1_ value, the $X_{1}^{\sigma }$ value was exhibiting any regular behavior (i.e., increase or decrease), but overall, their value was found to be higher at higher *α*_1_. The $X_{1}^{\sigma }$ value attained higher values in the NaCl system as compared with the aqueous solution at all *α*_1_, displaying that salt diminished the repulsive forces existing amid components. Accordingly, there was an affection for early micellization prompted via the progressively hydrophobic atmosphere.

The *β^σ^* values possess three possibilities: (1) *β^σ^* = 0 for an ideal monolayer, which means no interaction among the mixture ingredients, (2) *β^σ^* > zero for antagonistic interactions, whereas (3) *β^σ^* < zero signifies the supremacy of attractive or synergistic interactions amongst mixture ingredients.

The *β^σ^* values obtained was negative in all cases, revealing the existence of attractive interactions or synergism at the interfacial surface (Table 1) [26,27]. This occurred because of the closely packed formation of the mixed monolayer, owing to the clearly interactive forces amongst the ingredients at the interface in all utilized media. The decrease in electrostatic repulsion amongst molecules of both components applied its impact more at the planar interfacial surface, as compared in convex micelles [3]. The negative value of *β^σ^* was revealed interactions amongst the constituent allocated to ion-ion dipole as well as hydrophobic interactions irrespective of the employed media. Consequently, the merger of these forces overwhelmed all electrostatic repulsion amongst the ingredients. In NaCl or U media, the *β^σ^* value was not displaying a somewhat unique trend, but was found to be negative in the whole system (Table 1).

IMP+TX-100 mixtures display higher surface activity together with a much lesser *cmc* value as compared with individual IMP. The higher interaction amid the ingredients in the solution mixtures does not only serve as evidence for synergism in binary mixed system. Synergism in any mixed system at an interfacial surface occurs only if the subsequent circumstances are met [3]: (a) *β*^σ^ value should be below 0, and (b) $\left|\beta^{\sigma }\right|$ value should be more than ln(*C*_1_/*C*_2_) value, otherwise attractive mixed monolayers will be found. By viewing these results, it is shown that for all systems only first the circumstance was satisfied (Table 1) However, the second circumstance was not fulfilled in almost all cases. Therefore, attractive interactions were observed irrespective of the type of media employed for the surface tension reduction efficiency.

Akin to mixed micelles, the value of the activity coefficient of the employed ingredients (*f*_1_^σ^ (TX-100) and *f*_2_^σ^ (IMP)) at the boundary was also evaluated via subsequent equations [28]:

(8) $$f_{1}^{\sigma }=\exp\left[\beta^{\sigma }{\left(1-X_{1}^{\sigma }\right)}^{2}\right],$$

(9) $$f_{2}^{\sigma }=\exp\left[\beta^{\sigma }{\left(X_{1}^{\sigma }\right)}^{2}\right].$$

Table 1 shows that both *f*_1_^σ^ (TX-100) and *f*_2_^σ^ (IMP) values are obtained below one irrespective of the media employed [28]. Therefore, the system showed nonideal behavior as well as experienced attractive interactions amid the applied species at the boundary of the air-solvent. The results also showed that the *f*_2_^σ^ was found to be lower as compared to *f*_1_^σ^ (Table 1). This phenomenon showed that the involvement of IMP was much lower at the mixed monolayer than that of the TX-100. In NaCl or U media, no distinct behavior was detected.

### 2.3. Thermodynamic Parameters

Thermodynamic parameter, e.g., the Gibbs energy of adsorption ($\Delta G_{\mathrm{ad}}^{o}$) of the existing systems (pure and mixed), was obtained from Equation (10) [29,30]:

(10) $$\Delta G_{\mathrm{ad}}^{o}=\Delta G_{m}^{o}-\frac{\pi_{cmc}}{\Gamma_{max}}.$$

Table 2 showed the achieved $\Delta G_{\mathrm{ad}}^{o}$ value of pure and mixed systems in different media. For the calculation of $\Delta G_{\mathrm{ad}}^{o}$ of the current system, $\Delta G_{m}^{o}$ (Gibbs free energy) values were used from our previous article [18]. All $\Delta G_{\mathrm{ads}}^{o}$ values were negative, which was symbolic of the spontaneity of the adsorption process at the air-solvent interface and their magnitude were higher than those of the previously calculated $\Delta G_{m}^{o}$ value [18] of the corresponding system. The occurrence of $\Delta G_{\mathrm{ad}}^{o}$ > $\Delta G_{m}^{o}$ hypothesized that adsorption phenomena were favored over the association process, meaning that after finishing the adsorption process, the micellization process starts, i.e., a slight effort is required to complete this phenomenon (energy supplied in micellization to bring the monomers from the surface to micellar state). The $\Delta G_{\mathrm{ads}}^{o}$ value of the IMP+TX-100 mixture at all *α*_1_ of the surfactant was more negative than the value associated with an individual component (IMP and TX-100) (Table 2). These obtained results showed that the adsorption phenomenon was additionally feasible in case of a mixed monolayer, as compared with the monolayer formed by a singular component. The $\Delta G_{\mathrm{ads}}^{o}$ value did not view any specific trends in U or NaCl media in IMP+TX-100 mixtures. In the case of pure components, in NaCl/U media their negative value was found to increase/decrease, respectively.

One more thermodynamic parameter, named minimum free energy (*G_min_*), which is attained at the outmost adsorption at equilibrium, is also used to determine the attractive interaction/synergism at the interfacial boundary via Equation (11) [31,32].

(11) $$G_{min}=A_{min}\gamma_{cmc}N_{A}.$$

The value of the evaluated $G_{min}$ value is given in Table 2. The value of $G_{min}$ is usually correlated by the shipping of a component from the bulk system toward the interfacial boundary. The smaller magnitude of the $G_{min}$ value detected in any studied case was characteristic of intensified stability of the air-solvent boundary [3]. The level through which the $G_{min}$ value of the system is decreased is directly proportional to the extent of synergism allied through the system. The obtained $G_{min}$ in our case was found to be lower in magnitude, showing the thermodynamic stable air-solvent boundary. The $G_{min}$ seemed to be guileless in respect of any increase or decrease in value in any proper way by the occurrence of U/NaCl (Table 2).

An additional parameter of mixed monolayer called excess free energy ($\Delta G_{\mathrm{ex}}^{\sigma }$) of IMP+TX-100 was computed using Equation (12) [33,34,35,36].

(12) $$\Delta G_{\mathrm{ex}}^{\sigma }=RT\left[X_{1}^{\sigma }\ln f_{1}^{\sigma }+\left(1-X_{1}^{\sigma }\right)\ln f_{2}^{\sigma }\right].$$

**Figure 2:**
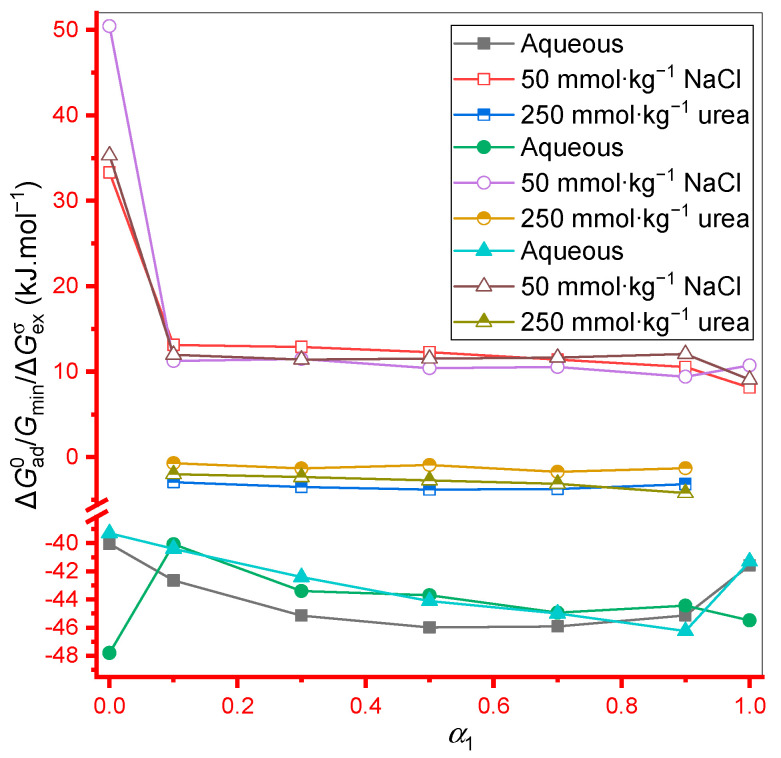
Variation of $\Delta G_{\mathrm{ad}}^{o}$/*G_min_*$/\Delta G_{\mathrm{ex}}^{\sigma }$ value with change in mole fraction (*α*_1_) of TX-100 in different media (filled, open, and half-filled symbols represent $\Delta G_{\mathrm{ad}}^{o}$, *G_min_*, and $\Delta G_{\mathrm{ex}}^{\sigma }$ respectively).

The obtained value of $\Delta G_{\mathrm{ex}}^{\sigma }$ was found to be negative in each solvent, observing that mixed monolayer formation is more stable than compared with a monolayer of either singular constituent (Table 2). Usually, at higher *α*_1_, the $\Delta G_{\mathrm{ex}}^{\sigma }$ value was found to be more negative, indicating that stability of the mixed monolayer was attained more at higher *α*_1_, however, the $\Delta G_{\mathrm{ex}}^{\sigma }$ value is not exhibiting a specific trend with the change of solvent (Table 2). Figure 2 showed the variation of $\Delta G_{\mathrm{ad}}^{o}$/*G_min_*$/\Delta G_{\mathrm{ex}}^{\sigma }$ value with change in mole fraction (*α*_1_) of TX-100 in different media (filled, open, and half-filled symbols represent $\Delta G_{\mathrm{ad}}^{o}$, *G_min_*, and $\Delta G_{\mathrm{ex}}^{\sigma }$ respectively) which depicted the comparison of different evaluated thermodynamic parameters graphically.

### 2.4. Packing Parameters

The structural geometry can be supposed via the packing parameter (*P*), i.e., the shape of micelles/mixed micelles in aqueous and non-aqueous solution was assessed through the following equation [37]:

(13) $$P=\frac{V_{0}}{A_{\min}l_{c}}.$$

In Equation (13), *l_c_* and $V_{0}$ are the effective chain length and volume of micellar interior, respectively, of the hydrophobic part of the employed monomers. Here, $A_{\min}$ value was used as achieved from the surface tension measurement. The $V_{0}$ and *l_c_* value were computed by employing Tanford’s theory [38].

(14) $$V_{0}=\left[27.4+26.9\;\left(n_{c}-1\right)\right]\times 2\;\left({Å}^{3}\right),$$

(15) $$l_{c}=\left[1.54+1.26\;\left(n_{c}-1\right)\right]\;\left(Å\right).$$

Here, *n*_c_ represents the whole sum of C-atoms in the C-chain length. The entire sum of C-atoms is measured one beneath the real count of C-atoms for the calculation of $V_{0}$ and *l_c_* value, since the C-atom next to the head group is extremely solvated. Hence, the first corban is also considered as the head group portion [3]. Table 2 depicted the evaluated *P* (packing parameter) value of the entire system. Micelles can be found in several shapes, depending on the obtained *P* value. As stated in literature [3,39] spherical micelles were detected as *P ≤* 0.333, cylinders or rods shapes micelles were noted for 0.333 < *P* < 0.5, vesicles and bilayers shapes micelles were found for 0.5 < *P* <1, whereas inverted micelles were reported for *P* > 1. In our case, a *P* value for IMP was obtained for 0.333 < *P* < 0.5 in the aqueous and U solvent, signifying that the micelles formed by IMP were cylinders or rods. In the NaCl solvent, the *P* value of IMP was found for 0.24, showing that IMP formed spherical micelles in the presence of NaCl (Table 2). For singular TX-100, the *P* was attained 0.5 < *P* < 1 irrespective of the employed solvent, representing that the micellar shape of TX-100 were vesicles (Table 2). For the IMP+TX-100 mixture of the different ratio in the presence of a different solvent, the *P* value was achieved 0.5 < *P* < 1, showing that a vesicle-shaped mixed micellar solution formed like pure TX-100, because mixed micelles consist of a maximum share of TX-100.

### 2.5. ^1^H NMR Study

**Figure 3:**
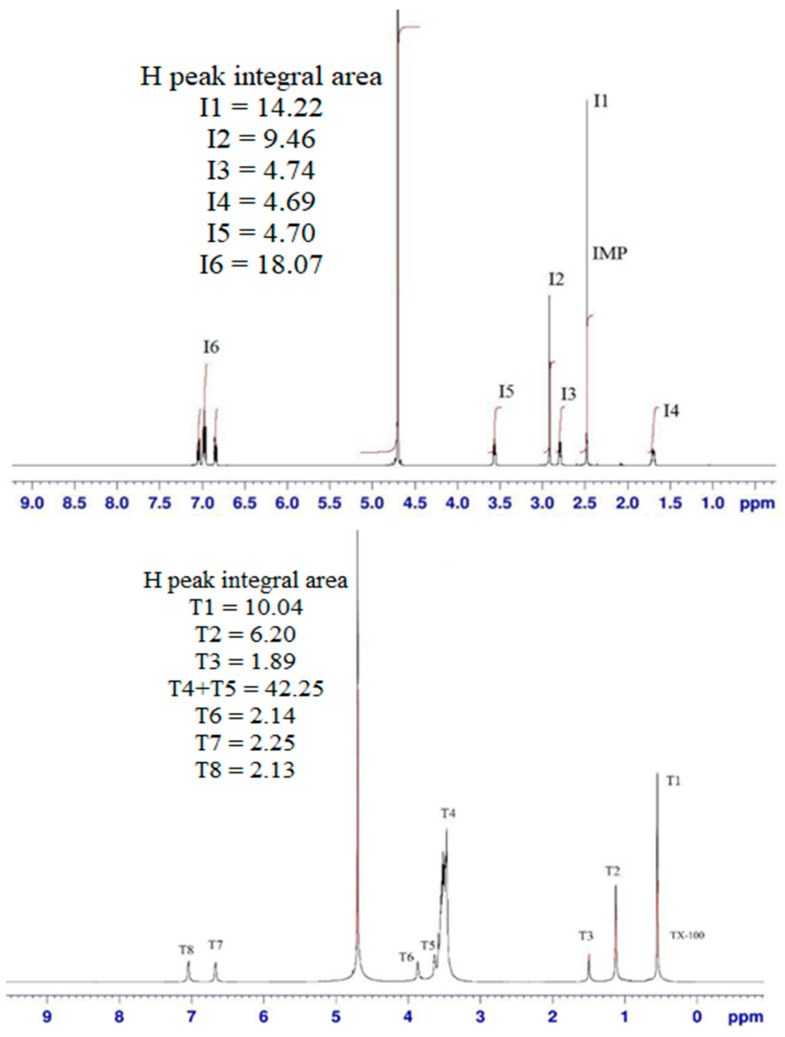
^1^H NMR (600 MHz) spectrum of singular compound IMP drug and TX-100 in D_2_O.

**Scheme 2:**
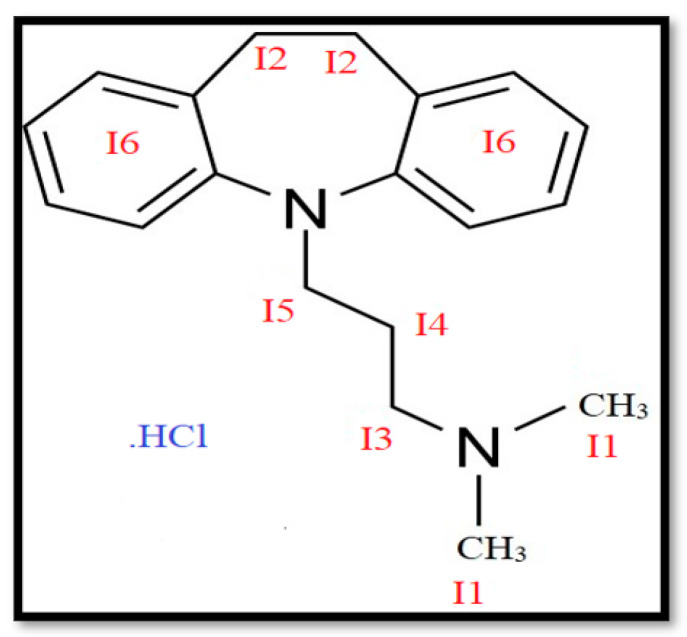
Molecular model of drug IMP.

**Scheme 3:**
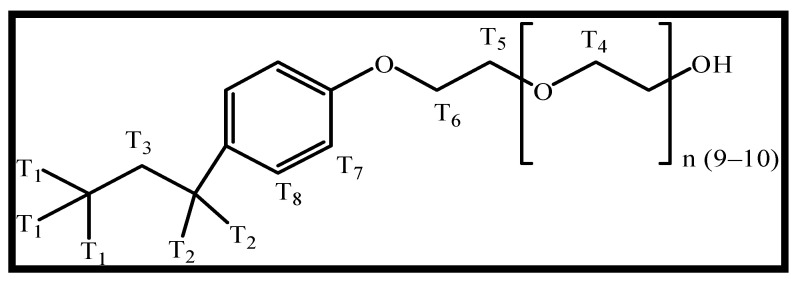
Molecular model of TX-100.

^1^H NMR technique is one of the finest methods for confirming the structure and purity of compounds [40,41]. Currently, ^1^H NMR is a very powerful method for examining an intermolecular interaction between both different compounds in their mixed micelles [42,43] and it gives us a great deal of information of interaction that is usually not available with other techniques. The present study also deals with the ^1^H NMR study of the interaction among the drug IMP and TX-100 surfactant in different ratios in their mixed micellar solution of an aqueous system. The ^1^H NMR signals of pure IMP, as well as TX-100, are clearly visible in D_2_O. The ^1^H NMR spectra of singular drug IMP and TX-100 is shown in Figure 3 with labeled hydrogen atoms attached to various carbons and obtained chemical shift value exposed in Table 3. Related data of pure TX-100 ^1^H NMR have also been given in previously published work [24,44]. The spectra of pure IMP clearly show distinct six proton signals, and their corresponding proton numbers are allotted in the structure given in Scheme 2. The pure TX-100 spectra clearly show eight proton signals and the corresponding proton numbers are allocated in Scheme 3 [24,44]. Protons attached to -N^+^(CH_3_)_2_ signals (I1 protons) are highly deshielded, that is, they resonate at high δ values because of the occurrence of N-atom in the drug IMP head group. All the NMR signals in both compounds drug IMP and non-ionic surfactant TX-100 (I1-I6 and T1-T8), in their pure form, show an increase in chemical shift δ values, which shows that each proton signal was highly deshielded. The proton signal I4 resonates at low δ values. This can be clearly observed, from the change in chemical shift values of I1, I3, I2, and I5, that the proton signals that present nearby to the head group are highly deshielded because of the occurrence of an adjacent N atom, whereas the proton signal I4 is highly shielded. No doubt, due to the combined electrostatic and hydrophobic effects, the interaction is stronger. In both drug and surfactant, the aromatic protons I6, T7, and T8 resonate at high δ values, i.e., they shift downfield.

**Figure 4:**
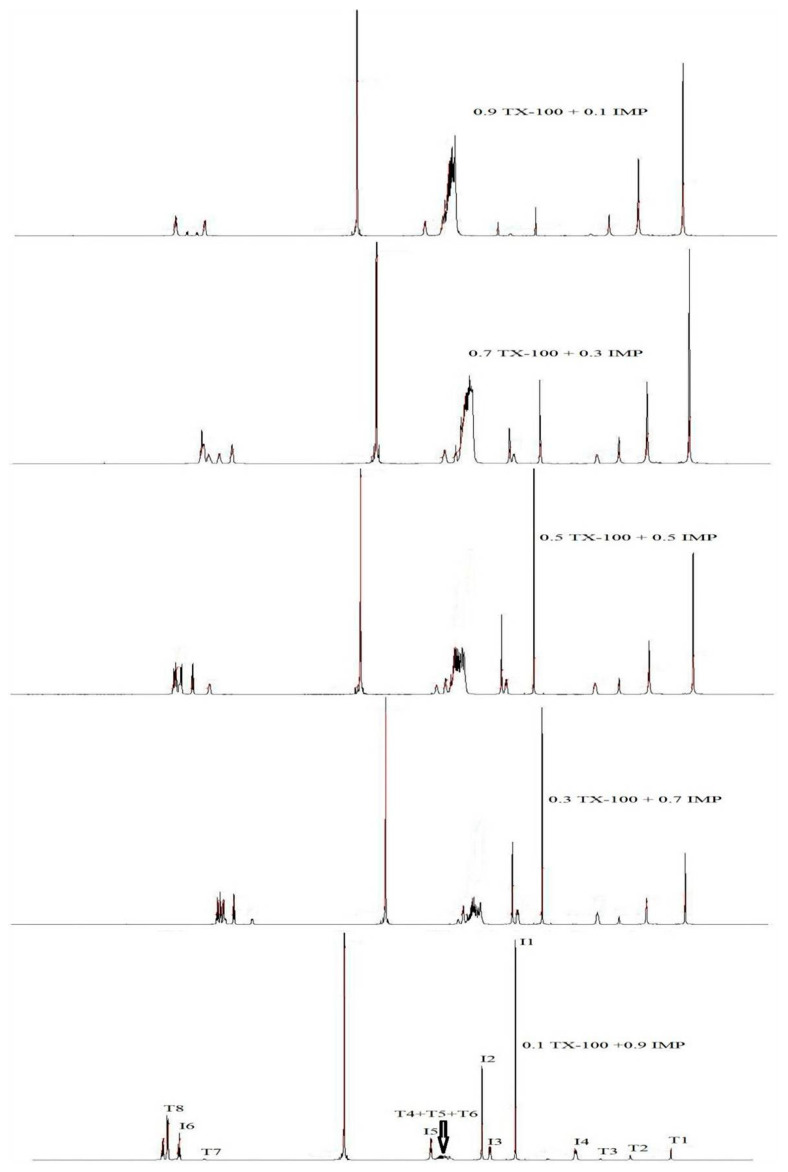
^1^H NMR (600 MHz) spectrum of IMP+TX-100 mixture having various *α*_1_ of TX-100 in D_2_O.

Substantiation of complex formation for the drug–surfactant mixtures was obtained by NMR spectroscopy [45]. The proton signals of both the drug and surfactant show a significant change upon mixing (IMP+TX-100), which can be clearly understood from the chemical shift values given in Table 4 and also from the spectra presented in Figure 4. Table 4 depicted the addition of TX-100 in pure IMP solution cause noteworthy displacement in chemical shift values, which clearly point towards molecular interaction between IMP and TX-100. Chemical shifts are used to describe signals in NMR spectroscopy and the location and number of chemical shifts is symbolic of the structure of a compound.

Upon addition of TX-100 to pure IMP, a slight increase in chemical shift values is seen, i.e., they show a downfield shift, but not much interaction is seen at lower mixing ratios i.e., 0.1 TX-100 and 0.3 TX-100. However, as the mole fraction of TX-100 reaches 0.5, a prominent enhancement in δ values is seen through a rise in mole fraction (0.5–0.9), and from these values, it can be concluded that the extent of downfield shift is caused by the addition of TX-100; this depends upon the *α*_1_ of surfactant in the solution of a drug and surfactant mixture. This increase in a downfield shift can be ascribed to an interaction of rigid tricyclic ring of IMP and polyoxyethylene chain of TX-100 structure.

The changes in chemical shift values for the alkyl protons I1 to I5 upon addition of TX-100 are also given in Figure 4 and Table 4 and it is clear that upon mixing of both studied constituents, the resultant mixed micelles cause deshielding (a downfield shift) of all the hydrophobic tail protons of IMP. Upon mixing, the hydrophobic interactions, as well as electrostatic attractions, endorse spherically along with the compacted micelles, while steric repulsion sources the hindrance amongst the constituents, causing the exposure along with protons deshielding. Overall, the proton signals (I1–I6, T1–T8) for both drug IMP and surfactant TX-100 in mixed micelles resonating at high δ values show a downfield shift of protons. It is known that both electrostatic, as well as steric interactions, show the leading character during the mixed micelles formation. Therefore, through the rise in TX-100 mole fraction, all proton signals for drug-surfactant mixtures are highly deshielded, which point towards an increase in steric repulsion among the molecules, which leads to the formation of large micelles [45,46]. As compared to pure IMP, the length of peak I4 is increased in case of mixtures up to mole fractions 0.5, but as the mole fraction reaches 0.7 and 0.9, the length of the signal I4 is decreased, which shows that these mole fractions (0.7 and 0.9) of TX-100 are more effective as compared to IMP. Similar changes were recorded for other NMR signals, such as I1. It is clearly visible from the spectra as well, being stable, that the aromatic protons related to the tricyclic rigid ring in IMP as well as the protons related to the mono aromatic ring in TX-100 are highly deshielded and show high δ values. Therefore, a clear downfield shift is observed. In the case of mixtures, all peaks are showing a clear downfield shift for both compounds, I1–I6 as well as T1–T8, at different mixing ratios. The compactness of the micelles varies with the variation of mole fraction, which is clear from the chemical shift values [47,48]. This change in chemical shift values is attributed to the interplay of electrostatic and steric interactions.

### 2.6. FT-IR Study

**Figure 5:**
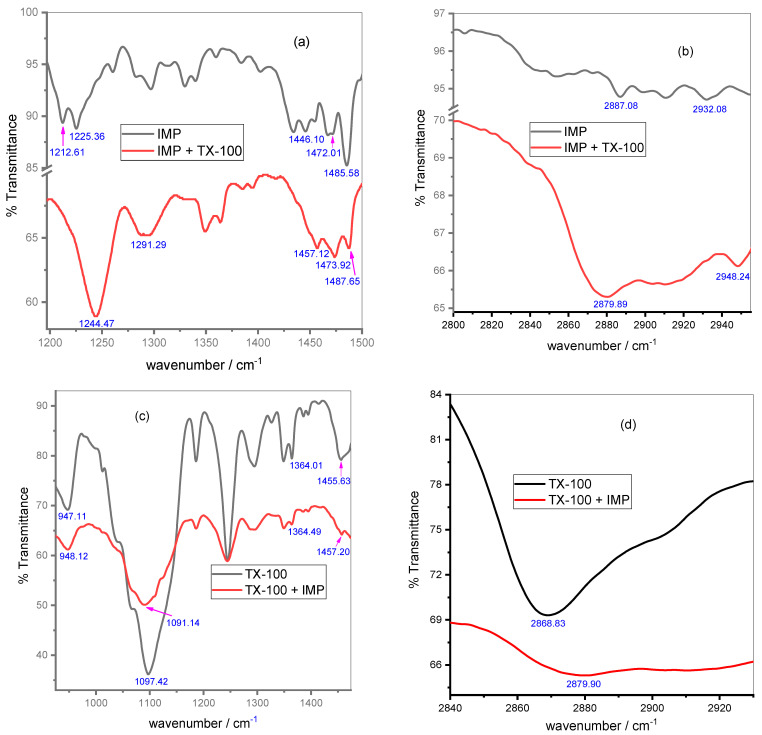
FTIR spectra of IMP (**a**,**b**) in the absence and existence of TX-100 and FTIR spectra of TX-100 (**c**,**d**) in the absence and existence of IMP in the selected wavenumber regions (cm^−1^).

The interaction impact can also be qualitatively followed via FT-IR spectra [49]. FT-IR spectroscopy was utilized to describe diverse functional groups and to examine the interaction amongst unlikely groups existing in the binary mixed system. Background-deducted FT-IR spectra of a pure drug and IMP+TX-100 mixed system of equal ratio in an aqueous solution are depicted in Figure 5a,b. Amphiphilic compound head-groups along with hydrophobic portion frequencies give statistics on the structural change in the monomers of formed micelles [50,51]. The feasible interaction amongst IMP+TX-100 mixed system will possibly alter the C–H bending and stretching and C–N stretching frequency of the drug head group.

To view the effect of TX-100 on the aliphatic C–N bond stretching band as well as C–H bond bending band in the IMP molecule of IMP+TX-100 mixture, a frequency range of 1195 to 1500 cm^–1^ was selected (Figure 5a). As shown from Scheme 2, the nature of the employed drug IMP is cationic, as it keeps a positively charged N atom allied with three alkyl groups. As depicted in Figure 5a, the singular IMP spectra showed C–N bond stretching at two different frequencies: one at 1212.61 and the second one at 1225.36 cm^–1^. However, in the occurrence of TX-100, the C–N stretching in IMP was shifted to a higher frequency. The first C–N bond stretching was shifted to 1244.47 from 1212.61 cm^–1^, and the second one was shifted to 1291.29 from 1225.36 cm^–1^. IMP showed C–H bending at three different frequencies (1446.10, 1472.01, and 1485.58 cm^–1^) (Figure 5a) and in the occurrence of TX-100, the frequency of C–H bending in IMP was significantly shifted to a higher frequency from their initial position (1457.12 cm^–1^, 1473.92 cm^–1^, and 1487.65 cm^–1^, correspondingly) because of the interaction of TX-100 with IMP. Shifting to a higher or lower frequency region is dependent on the environment of the interacting group of molecules. Through the addition of TX-100, the alteration in C–N stretching and C–H bending frequency in IMP showed the attractive interaction between constituents, owing to mixed micelles formation.

To investigate the C–H stretching in IMP, the frequency band region of 2800 to 2960 cm^–1^ was chosen to assess the effect of TX-100, and the achieved plotted graph is displayed in Figure 5b. As depicted in the graph, IMP showed a C–H bond stretching band at 2887.08 as well as 2932.08 cm^–1^ of the alkane methyl group. In presence of TX-100, the obtained C–H bond stretching band was shifted from 2887.08 to 2979.89 and 2932.08 to 2948.24 cm^–1^. In the presence of TX-100, the occurrence of this shifting in the C–H stretching frequency band in the IMP functional group, reveals an interaction amongst both employed ingredients (IMP and TX-100) [52].

Figure 5c,d depicted the FT-IR spectra of a singular TX-100 and TX-100+IMP mixture with an identical ratio. Figure 5c showed the spectra of singular TX-100 between 940 and 1470 cm^–1^ frequency that showed the C–O stretching at 947.11 and 1097.42 cm^–1^, O–H bending at 1364.01 cm^–1^, and C–H bending band at 1455.63 cm^–1^. Upon addition of IMP in the solution of TX-100, both C–O bond stretching was shifted from their original position. The first one shifted from 947.11 to 948.12 cm^–1^, and the second one shifted from 1097.42 to 1091.14. In addition, in the presence of IMP, the shifting in the frequency band of O–H bending in TX-100 occurred from 1364.01 to 1364.49 cm^–1^ and the C–H bending band from 1455.63 to 1457.20 cm^–1^, signaling the interaction amongst the constituents. Figure 5d showed the spectra of TX-100 as well as the TX-100+IMP mixture in the range 2840–2930 cm^–1^. Pure TX-100 showed the medium and broad C-H stretching band (alkane) at 2868.83 cm^–1^. The C–H stretching band (alkane) attained at 2868.83 cm^–1^ in TX-100 was moved to a higher frequency (2879.98 cm^–1^) in the presence of IMP, signifying an interaction amongst TX-100+IMP mixture mixed micelles. The O–H stretching band was found in case of singular TX-100, but for the TX-100+IMP mixture, the O–H stretching band peak disappeared due to merging with the water peak (not shown graphically). Due to the interaction of the employed ingredients, the whole frequency band variation did not achieve much, but obtained to be reproducible. Overall, herein, the shifting in C–N stretching, O–H bending, along with C–H bending, and stretching frequency recommend the interaction between the employed ingredients [53,54,55].

## 3. Conclusions

Before a surfactant can be employed as an appropriate drug agent, a broad range analysis must be accomplished to examine the interaction of the surfactant through the proposed drug. Herein, ^1^H NMR, FT-IR, and tensiometric studies were performed to explore the interaction of a TX-100 surfactant with the cationic drug IMP. Physiologically, the nonionic nature of surfactants is more suitable as compared with ionic ones (cationic/anionic) and, owing to their high surface activity, a nonionic surfactant, such as TX-10, is considered as an ideal nominee for drug delivery in comparison to other surfactants. The interfacial properties of IMP, TX-100 along with the IMP+TX-100 mixture of various ratios at the surface were evaluated using a tensiometric method in different solvents (H_2_O/NaCl/urea). TX-100 decreases the surface thickness acquired by means of the water layer and enhances the hydrophobic film width of the studied systems. Interfacial composition ($X_{1}^{\sigma }$) and the *β*^σ^ values of the IMP+TX-100 system showed a much higher participation of TX-100 at the surface than IMP and attraction/synergism between the components at the surface, respectively. The obtained value of $\Delta G_{\mathrm{ad}}^{o}$ specifies that the adsorption phenomena was a spontaneous process and the stability of the mixed monolayer. The *P* value of IMP+TX-100 was attained as 0.5 < *P* < 1, showing that the micellar solution was vesicles-shaped. The value of *Γ_max_* acquired more for the surfactant than IMP, confirming that the surfactant showed higher surface-activity as monomers of TX-100, favoring a compacted or strongly packed arrangement at the surface in all solvents. ^1^H NMR study of solution mixed systems advocated that IMP and TX-100 interact with each other via hydrophobic interaction. FT-IR spectra showed that the frequency band of individual ingredients (IMP and TX-100) was shifted from the original position for the mixed system, proving the interaction amongst them. The conclusions of the current investigation contribute to the assessment of the implementation of the surfactant (as a capable drug delivery) with a drug mixed system and the supporting mechanisms, for a basic understanding required for the projected expansion of economical and efficient drug formulations.

## 4. Materials and Methods

### 4.1. Materials

Every material in the current study was used as received from their respective company. Drug IMP was obtained from Sigma (St. Louis, MO, USA) having purity ≥ 98.0%. Surfactant TX-100 was from Sigma (Taufkichen, Germany). Different additives such as NaCl was acquired from BDH (Poole, England), having a purity of 98.0%, and urea was obtained from Sigma (Taufkichen, Germany), with a purity of 98.0%. Deuterium oxide (D_2_O) was purchased from Sigma (St. Louis, MO, USA) with a purity of 99%, which was used as the solution preparation for the ^1^H NMR study only. For the rest of the study, distilled water was used for the solution preparation. Using calculated quantities of NaCl and urea dissolved in distilled water, the prepared solutions of these additives were used as solvents. In the aqueous system and in the occurrence of fixed NaCl/urea concentrations, the stock solutions of both employed constituents (IMP and TX-100) of fixed concertation were made separately, clearly above their corresponding *cmc*. Combinations of both components (IMP (drug) and TX-100 (surfactant as drug carrier) were readied by mixing the prepared stock solutions of both constituents (IMP and TX-100) in diverse mass ratios, varying the mole fraction of component 1 (TX-100 surfactant) from 0.1 to 0.9. These prepared solutions of diverse mass ratios were employed in the experiments, assuming that the density of the component’s dilute solution at the experimental temperature is roughly constant.

### 4.2. Methods

#### 4.2.1. Measurement of Surface Tension

For the surface tension (*γ*) measurement, an Attension tensiometer (Sigma 701, Darmstadt, Germany) working with the ring detachment process was applied for pure (IMP and TX-100) and mixed system (IMP+TX-100) in five ratios in aqueous/NaCl/U solvent. The *γ* of resultant system (IMP, TX-100, or IMP+TX-100) vs. *log* (*C* (conc.)) of pure IMP, TX-100, or IM+TX-100 were plotted, and each plot showed a break point that was termed *cmc* of the system [18]. Here, plots given for the IMP+TX-100 mixed system in different media in our previous work [18] were used for evaluation of different interfacial parameter evaluation. The error in *γ* and temperature was attained as ± 0.2 mNm^−1^ and ± 0.2 K, respectively.

#### 4.2.2. ^1^H NMR Study

For the ^1^H NMR study, D_2_O (as a solvent) was used rather than distilled water to prepare the solutions of the individual component (IMP, TX-100) and their mixtures (IMP+TX-100). The ^1^H NMR spectra of IMP, the surfactant, and their mixture of various mole fractions in the aqueous system, were noted using a Bruker ultrashield plus 600 spectrometer, Billerica, MA, USA (600 MHz proton resonance frequency). Approximately 1 mL of every studied system is placed in a 5 mm tube for spectra measurements and chemical shifts were noted on the *δ* (ppm) scale. The reproducibility of *δ* was within 0.01 ppm. An organosilicon compound-tetramethylsilane was employed as an internal standard, which is recognized for calibrating a chemical shift.

#### 4.2.3. FTIR Spectroscopy

In the aqueous system, the FTIR spectra (4000 to 400 cm^–1^ wavelength) of the singular components and IMP+TX-100 mixed system in an equal ratio were recorded by consuming a NICOLET iS50 FT-IR spectrometer possessing ATR accessory (Thermo Scientific, Madison, Waltham, MA, USA). Here, a particular part of the wavelength range is exposed in the graph for clarity purposes. From the entirely attained spectra of the chosen system, the water spectrum was consistently deducted. The concentration of IMP and TX-100 was maintained very well above their respective *cmc* value. Each spectrum was obtained at a resolution of 4.0 cm^−1^.

## Figures and Tables

**Table 1:** Different interfacial parameters for IMP+TX-100 mixture in several media at 298.15 K ^a^.

*α*_1_	*X*_1_^σ^	*β^σ^*	*f_1_^σ^*	*f_2_^σ^*	*Γ_max_* 10^7^ (mol·m^−2^)	*A_min_*/*A^id^* (Ǻ^2^)	*γ_cmc_* (mN·m^−1^)	*π_cmc_* (mN·m^−1^)	*pC*_20_	ln(*C*_1_/*C*_2_)
Aqueous solution										
0					12.78	129.95	42.58	28.42	1.95	
0.1	0.7334	−6.07	0.6496	0.0382	23.09	71.89/137.77	30.35	40.65	3.90	−6.04
0.3	0.7621	−7.77	0.6443	0.0110	23.22	71.51/139.94	29.94	41.06	4.36	−6.04
0.5	0.7741	−8.79	0.6385	0.0052	24.16	68.72/140.84	29.67	41.33	4.58	−6.04
0.7	0.7969	−9.34	0.6803	0.0027	25.73	64.54/142.56	29.36	41.64	4.68	−6.04
0.9	0.8421	−9.62	0.7868	0.0011	27.90	59.52/145.97	29.40	41.60	4.71	−6.04
1					36.02	46.10	29.31	41.69	4.57	
50 mmol∙kg^−1^ NaCl										
0					8.86	187.41	44.69	26.31	2.07	
0.1	0.8828	−2.76	0.9629	0.1168	27.25	60.93/149.04	30.66	40.34	3.88	−6.32
0.3	0.8712	−4.80	0.9235	0.0262	26.04	63.77/148.16	29.91	41.09	4.38	−6.32
0.5	0.9149	−4.76	0.9661	0.0186	28.65	57.95/151.46	29.83	41.17	4.57	−6.32
0.7	0.8819	−6.75	0.9101	0.0052	28.01	59.27/148.97	29.53	41.47	4.75	−6.32
0.9	0.9210	−7.23	0.9559	0.0022	31.30	53.05/151.91	29.45	41.55	4.82	−6.32
1					27.60	60.15	29.65	41.35	4.96	
250 mmol∙kg^−1^ U										
0					12.46	133.24	44.03	26.97	1.86	
0.1	0.7776	−4.69	0.7930	0.0587	25.19	65.91/141.11	30.21	40.79	3.70	−6.05
0.3	0.8085	−6.12	0.7989	0.0183	25.92	64.06/143.43	29.59	41.41	4.15	−6.05
0.5	0.8149	−7.26	0.7797	0.0080	25.42	65.31/143.92	29.31	41.69	4.38	−6.05
0.7	0.8189	−8.50	0.7567	0.0033	25.57	64.94/144.22	29.80	41.20	4.54	−6.05
0.9	0.8096	−10.99	0.6715	0.0007	24.60	67.49/143.52	29.71	41.29	4.71	−6.05
1					33.18	50.04	30.17	40.83	4.49	

^a^*A*_1_ = *A_min_* of TX-100 and *A*_2_ = *A_min_* of IMP. *A*_1_ = 46.10 (in aqueous), 60.15 (in NaCl), 50.04 Ǻ^2^ (in urea). *A*_2_ = 129.95 (in aqueous), 187.41 (in NaCl), 133.24 Ǻ^2^ (in urea).

**Table 2:** Various thermodynamic parameters along with packing parameter (*P*) for pure and IMP+TX-100 mixture in various media.

*α*_1_	$\Delta G_{\mathrm{ad}}^{o}$ (kJ·mol^−1^)	*G*_*min*_ (kJ·mol^−1^)	$\Delta G_{\mathrm{ex}}^{\sigma }$ (kJ·mol^−1^)	*P*
Aqueous system				
0	−40.06	33.33		0.34
0.1	−42.66	13.14	−2.94	0.60
0.3	−45.15	12.89	−3.49	0.61
0.5	−45.99	12.28	−3.81	0.63
0.7	−45.92	11.41	−3.74	0.67
0.9	−45.13	10.54	−3.17	0.73
1	−41.58	8.14		0.95
50 mmol∙kg^−1^ NaCl				
0	−47.80	50.44		0.24
0.1	−40.08	11.25	−0.71	0.71
0.3	−43.40	11.49	−1.33	0.68
0.5	−43.70	10.41	−0.92	0.75
0.7	−44.94	10.54	−1.74	0.73
0.9	−44.45	9.41	−1.30	0.82
1	−45.48	10.74		0.73
250 mmol∙kg^−1^ U				
0	−39.30	35.33		0.33
0.1	−40.40	11.99	−2.01	0.66
0.3	−42.41	11.42	−2.35	0.68
0.5	−44.11	11.53	−2.71	0.66
0.7	−45.0	11.66	−3.12	0.67
0.9	−46.25	12.08	−4.20	0.64
1	−41.30	9.09		0.87

**Table 3:** ^1^H NMR chemical shifts (δ, ppm) of pure IMP and TX-100 in aqueous system.

Compound	Chemical Shifts (δ, ppm)							
Pure IMP	I1	I2	I3	I4	I5	I6		
	2.478	2.916	2.790	1.695	3.564	6.983		
Pure TX-100 ^a^	T1	T2	T3	T4	T5	T6	T7	T8
	0.549	1.124	1.495	3.591	3.806	3.865	7.015	7.097

^a^ References [24,44].

**Table 4:** ^1^H NMR chemical shifts (δ, ppm) of IMP+TX-100 mixtures in aqueous system.

	Chemical Shifts (δ, ppm)				
	*α*_1_ = 0.1	*α*_1_ = 0.3	*α*_1_ = 0.5	*α*_1_ = 0.7	*α*_1_ = 0.9
T1	0.486	0.517	0.527	0.534	0.542
T2	1.01	1.06	1.08	1.096	1.111
T3	1.376	1.389	1.437	1.464	1.473
T4–T6	3.406	3.417	3.42	3.44	3.517
T7	6.82	6.831	6.944	6.999	7.004
T8	6.975	6.997	7.007	7.026	7.029
I1	2.496	2.515	2.524	2.526	2.685
I2	2.924	2.93	2.932	2.937	3.41
I3	2.834	2.845	2.86	2.874	2.903
I4	1.716	1.754	1.759	1.764	1.817
I5	3.57	3.582	3.596	3.628	3.65
I6	6.991	6.998	7.012	7.018	7.024

## Data Availability

Not applicable.
